# Immunotherapy‐Related Gastritis in Small Cell Lung Cancer Treatment With Durvalumab—A Case Report

**DOI:** 10.1002/cnr2.70422

**Published:** 2025-12-02

**Authors:** Marc C. Cavaliere, Silvana Spadafora, Michela Febbraro

**Affiliations:** ^1^ McMaster University Hamilton Canada; ^2^ Algoma District Cancer Program Sault Ste Marie Canada

**Keywords:** durvalumab, gastritis, immune checkpoint inhibitor, immune‐related adverse event, immunotherapy, small‐cell lung cancer

## Abstract

**Background:**

Durvalumab is a PD‐L1 inhibitor that triggers a blockade resulting in enhanced anti‐tumor responses related to increased T‐cell activation. There is potential for numerous immune‐related adverse events (irAEs) with this treatment, most of which have been shown to be effectively managed with high‐dose steroids. Immunotherapy‐related gastritis, while rare compared to other irAEs is an emerging concern as the use of immune checkpoint inhibitors (ICIs) increases.

**Case:**

This case study examines a 66‐year‐old female with extensive‐stage small cell lung cancer (ES‐SCLC) treated with durvalumab, alongside chemotherapy. Twenty‐three months into treatment, she developed non‐specific gastrointestinal (GI) symptoms including abdominal pain, appetite loss, and significant weight loss. Despite conservative management, resolution only occurred following the use of high‐dose steroids, a finding consistent with immunotherapy‐related gastritis. The patient then went onto a successful rechallenge of immunotherapy.

**Conclusion:**

This case represents the first report on the rare occurrence of immune‐related gastritis in an ES‐SCLC patient who has been on immunotherapy for nearly 2 years. Current literature is limited in the understanding of underlying mechanisms of PD‐L1‐related irAEs and optimal management strategies for rare toxicities like gastritis in immunotherapy‐treated cancer patients. This report aims to address the unmet need for further research on rare toxicities to immunotherapy in unique cases.

## 1 Background

Immunotherapy‐related gastritis is relatively rare compared to other gastrointestinal irAEs such as colitis or hepatitis [1]. In fact, there are currently no publications reporting on a SCLC patient developing irAE gastritis after over 1 year of immunotherapy treatment. SCLC is an aggressive malignancy characterized by rapid growth, high metastatic potential, and poor overall prognosis. Tumor cells in SCLC express programmed death‐ligand 1 (PD‐L1) as an immune escape mechanism [2]. This immune suppression is particularly significant in patients with ES‐SCLC, a condition whereby the cancer has spread beyond a single lung and is incurable [3]. Studies by Xagara et al. [2] have shown that SCLC patients exhibit higher percentages of PD‐1 expressing CD3^+^, CD4^+^, and CD3^+^CD8^+^ T‐cells, along with elevated PD‐1 expression compared to healthy individuals. However, compared to non‐small cell lung cancer (NSCLC), PD‐L1 expression in SCLC is both rarer and lower [4, 5]. Despite this, PD‐1/PD‐L1 inhibition has efficacy in the treatment of SCLC and its use in the incurable setting is standard of care [6].

Durvalumab blocks the interaction of PD‐L1 on tumor cells with PD‐1 and CD‐80 receptors on T‐cells [7]. This blockade prevents the initiation of immune‐inhibitory signals within activated T‐cells, enhancing anti‐tumor responses, which may be related to increased T‐cell activation [8]. The patient in this study was treated following the CASPIAN study [9]. In this clinical trial, 537 patients either received treatment with durvalumab immunotherapy plus platinum‐etoposide chemotherapy, or platinum‐etoposide chemotherapy. Durvalumab plus platinum‐etoposide significantly improved overall survival in patients with ES‐SCLC versus a clinically relevant control group [9].

While ICI treatments have significant clinical benefits, they can also trigger irAEs. These events are typically caused by the upregulation of the immune system and commonly include dermatitis, pneumonitis, thyroiditis, and diarrhea, among others [8]. Fortunately, such events are usually mild and can be effectively managed with high‐dose steroids [8]. The following case report presents the case of a patient with ES‐SCLC, who, following treatment using durvalumab, developed immunotherapy‐related gastritis a remarkable 23 months after the therapy was initiated. This case stands out due to the unusual timing of the adverse reaction, which occurred well into treatment, as well as the patient's uncommon diagnosis of SCLC.

## 2 Case Presentation

A 66‐year‐old female presented with sudden onset bone pains to her skull, neck and lower back in August 2021. Initial imaging completed shortly after at Sault Area Hospital, including CT, SPECT, and bone scans, along with an MRI of the spine, revealed lung nodules, and diffuse bone lesions along the axial skeleton and skull. The patient was diagnosed with extensive‐stage small cell lung cancer on lung biopsy. Given concerns of bony instability and risk for spinal cord involvement, she underwent palliative external beam radiotherapy to the skull and spine in November 2021. In December 2021, systemic therapy was initiated with a chemoimmunotherapy regimen of carboplatin, etoposide, and durvalumab. This included intravenous (IV) carboplatin and etoposide chemotherapy every 3 weeks for four cycles. After completing induction chemo‐immunotherapy in March 2022, she went onto maintenance durvalumab immunotherapy at a dose of 1500 mg IV every 4 weeks [10]. The introduction of systemic therapy was tolerated well with no significant side effects.

Relevant comorbidities included hypertension, chronic obstructive pulmonary disease (COPD), controlled gastroesophageal reflux disease (GERD), coronary artery disease, and sigmoid diverticular disease. The patient had a 60‐pack‐year smoking history; she was independent and working at the time of diagnosis with an ECOG performance status of zero.

**FIGURE 1:**
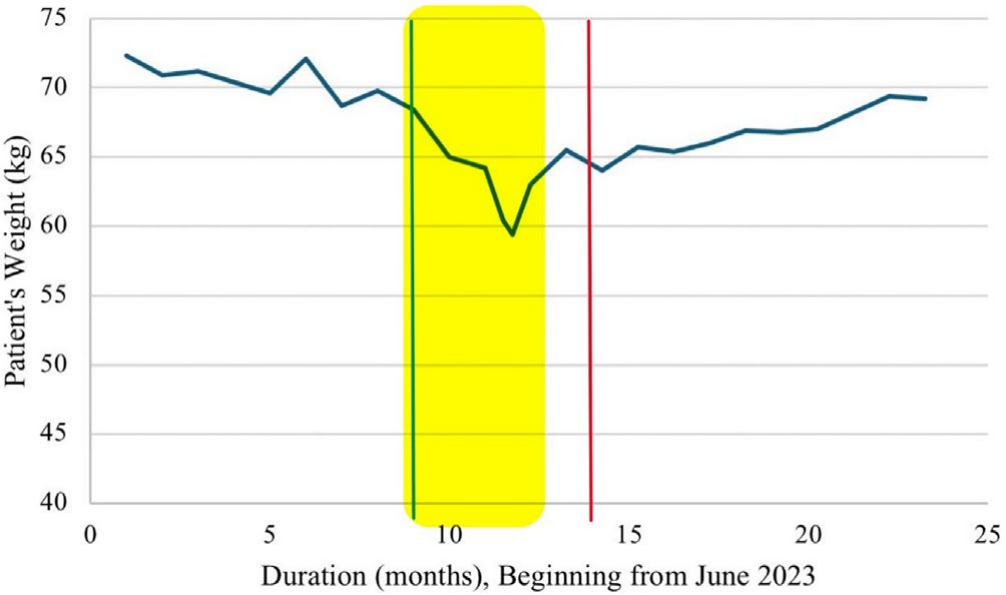
Graph depicting the patient's weight in kilograms over time in months. Time = 0 represents June 2023. Yellow highlight represents period of gastritis symptom presentation, green line represents when durvalumab was stopped, and red line represents when durvalumab was reintroduced after symptom resolution.

Maintenance durvalumab treatment continued with minimal interruptions. Throughout this time, the patient's regular surveillance imaging reported stable disease. In February 2024, the patient presented to the emergency department with recurrent abdominal and epigastric pain, which was exacerbated following eating, along with cramping and decreased appetite. There were no complaints of nausea or vomiting. Ultrasound revealed a dilated common bile duct (1.5 cm); however, this was deemed secondary to cholecystectomy rather than her symptoms as determined by MRCP. The MRCP also ruled out biliary stone(s) as the cause. The patient was referred to gastroenterology for endoscopy. Given the symptoms, the patient feeling unwell and a change in her ECOG performance status to two, durvalumab was placed on hold. While waiting for a GI consultation, the patient was managed with calcium carbonate antacids, milk of magnesia, histamine‐2 blocker and proton pump inhibitor. While awaiting endoscopy the patient's symptoms worsened. Despite steroid‐sparing measures, the patient's symptoms minimally improved, and she lost approximately 11.8 kg at the peak of her symptoms (Figure 1). As such, she was given prednisone 20 mg daily for 1 week, followed by a slow taper at 5 mg per week thereafter. This led to complete resolution of her symptoms after 14 days alongside a very liberal diet, with subsequent weight gain and improved appetite. Upper endoscopy was completed within the week of starting steroids which reported significant gastritis localized to the stomach by visualization. This included diffuse visible inflammation throughout the entire stomach without evidence of ulcers, tears, or erosions. The findings were in keeping with diffuse gastritis. Unfortunately, as Sault Area Hospital is a small center, endoscopy was completed off‐site and images are unavailable for further review. Despite visual findings of gastritis, the biopsy results were negative for inflammation or signs of active gastritis. These findings can be attributed to the delay in obtaining the biopsy, during which time steroid therapy likely resolved the inflammation. The pathology was also negative for infection or lymphocytic infiltration. The biopsy was negative for dysplasia and malignancy.

**FIGURE 2:**
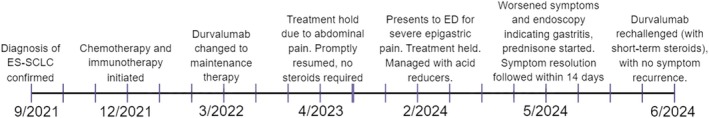
Timeline depicting some of the most significant events during the patient's course.

Durvalumab was restarted on June 19, 2024. With the administration of each cycle of durvalumab, 4‐day courses of prednisone 5 mg daily were administered to prevent symptom recurrence, which has proven effective. Over the following months, the patient experienced mild, tolerable GI symptoms that resolved spontaneously. A visual timeline depicting the most significant events during the patient's course can be seen in Figure 2. As of November 2025, the patient continues to be on durvalumab. She has not had any recurrence of her symptoms after complete response to and discontinuation of steroids. She continues to have a response to treatment with no evidence of disease progression on regular CT and bone scan surveillance.

## 3 Discussion

This case demonstrates the uncommon irAE of gastritis during immunotherapy with durvalumab. This is the first published case of immunotherapy‐related gastritis in a patient with ES‐SCLC who has been on immunotherapy for over 1 year. This case challenges existing assumptions that irAEs primarily occur early and suggests a broader time frame for potential adverse effects. GI symptoms first emerged nearly 2 years into immunotherapy treatment, and persisted for approximately 2 months.

The physicians attributed the gastritis to ICI therapy as a diagnosis of exclusion. Although the patient experienced mild reflux and GERD symptoms during cytotoxic therapy, as is often seen in patients affected by malignancy, these were very well controlled and notably different from the intensified symptoms observed during the gastritis episode. As well, alternative causes were ruled out in consult with gastroenterology. Given this, along with the positive response to steroid treatment, ICI‐induced gastritis is left as the most likely cause.

The prolonged time to symptom resolution can be largely attributed to a lack of awareness about this rare irAE. The unusually delayed onset of toxicity meant that immunotherapy‐related gastritis was not immediately suspected, complicating efforts to properly treat the patient. Limited industry knowledge and scarce literature on this condition led to additional testing and outgoing referrals which ultimately resulted in the delaying of the patient's treatment. The lack of characteristic endoscopic findings for irAE gastritis has led to its difficulty in management. This rare case exemplifies that physicians may have to treat symptomatically and not pathologically. To place this in a broader context, this case exemplifies the risk posed by rarer irAEs which remain under‐researched and lack evidence‐based treatment guidelines. Addressing these knowledge gaps through further research is crucial to identifying and managing rare irAEs more effectively. Although this case is distinct and not as widely generalizable, the lack of existing research highlights the adaptability required from physicians when treating rare and complex conditions.

The incidence of gastritis and/or abdominal pain has been less described than other toxicities associated with durvalumab [11]. In a controlled study of the safety and efficacy of durvalumab, 63.9% of patients experienced an irAE of any grade, the most common being fatigue (13.1% of patients), diarrhea (9.8%), and decreased appetite (8.2%) [12]. ICI‐related gastritis was noted to have an incidence of 0.35%–1.46% [13]. Additionally, it was found that the median time from ICI initiation to onset of gastritis was 3.4 months, with the range being a vast 0.3–109.4 months [13]. Of relevance to the patient in this study is decreased appetite, which she experienced—between the first presentation of abdominal pain and the beginning of her symptom improvement, she had lost 11.8 kg. This can be classified as its own adverse event, or secondary to her abdominal pain, which was often exacerbated by diet, or likely a combination of these factors.

After symptom presentation, our patient was treated with prednisone, an antacid, an H2 blocker, a PPI, along with the suspension of her immunotherapy. Though there are no gastritis‐specific management guidelines, Cancer Care Ontario (CCO) guidelines for similar GI toxicities (such as diarrhea/colitis) recommend referral to a gastroenterologist, steroid treatment, and dietary adjustments [14]. It was also discovered that after treatment with solumedrol, followed by a prolonged prednisone taper, the recurrence of symptoms was low (13%), suggesting that the recurrence risk is low with the appropriate treatment [15]. Unique to this case, is the lack of inflammatory features of her gastritis. Other cases in the literature, such as Goel et al. [11] explain thickening of the stomach consistent with inflammation, along with pathology which supports this. That being said, a systematic review by Su et al. [16] found that pathological examinations revealed lymphocyte infiltration in only 67.1% of patients. This reflects the lack of inflammatory features in the case of our patient and further indicates a need for physicians to treat clinically, rather than pathologically. Despite this, the resolution with steroid treatment signified that the symptoms were likely immunotherapy‐related.

To place the findings of our patient into the context of the current research of immunotherapy‐induced gastritis, current literature on the subject was reviewed. Three systematic reviews [13, 16, 17] looking at immunotherapy‐related gastritis across all cancer types were assessed, across a total of 337 patients. It was found that gastritis was noted in melanoma (38.6%) and NSCLC (34.7%) the most. As well, the most common presenting symptoms included nausea and vomiting (62%), abdominal pain (48%), and weight loss (30%). The timing of symptom onset varied greatly. The median onset was 5.6 months; however, this ranged from 0.5 to 39 months. SCLC was not discussed in these studies, and there is currently no literature that details ICI‐induced gastritis exclusively in SCLC cases.

Following this, a review of case studies pertaining to immunotherapy‐related gastritis exclusively in non‐small cell lung cancer (NSCLC) patients was conducted. In analyzing 16 patients, the most common symptoms were noted to be epigastric pain (68.8%), anorexia (50.0%), and nausea (43.8%). Steroids were used as treatment for 13 patients (81.3%), which in nearly all cases prompted symptom resolution [1, 18, 19, 20, 21, 22, 23, 24, 25]. In lung cancer cases, the time to onset was notably longer compared to other cancers, at 9.7 months. However, there was great variation in onset, ranging from 3 weeks to 48 months. Our patient, though diagnosed with SCLC and not NSCLC, follows these trends with her presenting symptoms, as well as the late onset in her symptoms. Few of these reports describe the reasoning and/or mechanisms behind the gastritis; however the most notable findings include the predominance of CD4^+^ and CD8^+^ T lymphocytes [18, 19, 21]. As well, previous research has drawn similarities to the pathogenesis of inflammatory bowel disease, specifically in similar immune activation patterns [18]. Despite a lack of knowledge on the pathways leading to ICI‐gastritis, extensive emphasis is placed on attaining a prompt biopsy for a definitive diagnosis.

The mechanism underlying the development of these irAEs remains relatively unclear to researchers. Further investigation is needed to explore the connection between PD‐L1 expression and irAEs [26]. Current hypotheses suggest that PD‐L1 expression in the stomach may contribute to the onset of irAE gastritis. By inhibiting the interaction between PD‐1 and PD‐L1, immune checkpoint inhibitors cause a blockade of immune tolerance, leading T‐cells to target antigens on gastric epithelial cells [26]. This emerging hypothesis requires validation through additional controlled studies. Although tissue‐based biomarkers like PD‐L1 have been linked to ICI effectiveness, their relationship to adverse events remains unclear, underscoring the importance of research into irAE‐specific pathogenesis [27].

## 4 Conclusion

This case details a female treated with durvalumab who developed immunotherapy‐related gastritis after 23 months of treatment, uniquely highlighting the difficulty in diagnosing and managing a rare irAE so far into treatment. Physicians should recognize the development of epigastric pain as one of the most common symptoms of irAE gastritis which will help promote proper treatment and increased success rates among cancer patients. Emphasis should be placed on the importance of being able to recognize and appropriately treat rare irAEs, like gastritis, to the same extent as other irAEs regardless of the timing of symptom onset. Rarer irAEs, such as gastritis are largely under‐researched. Mechanisms of irAE‐specific pathogenesis require close study for both common and uncommon events.

## Author Contributions

Conceptualization: Michela Febbraro. Data curation: Marc C. Cavaliere and Michela Febbraro. Writing – original draft: Marc C. Cavaliere. Writing – review and editing: Michela Febbraro and Silvana Spadafora. Visualization: Marc C. Cavaliere. Supervision: Michela Febbraro and Silvana Spadafora.

## Funding

The authors have nothing to report.

## Ethics Statement

The authors declare that the manuscript has been created in accordance with Wiley's Publication Ethics Guidelines, in an ethical and responsible way, with no research misconduct.

## Consent

Informed consent was obtained from the patient involved in the study.

## Conflicts of Interest

The authors declare no conflicts of interest.

## Data Availability

The data that support the findings of this study are available on request from the corresponding author. The data are not publicly available due to privacy or ethical restrictions.
